# Lipids and pathogenic flaviviruses: An intimate union

**DOI:** 10.1371/journal.ppat.1006952

**Published:** 2018-05-10

**Authors:** Hans C. Leier, William B. Messer, Fikadu G. Tafesse

**Affiliations:** 1 Department of Molecular Microbiology & Immunology, Oregon Health & Science University (OHSU), Portland, Oregon, United States of America; 2 Department of Medicine, Division of Infectious Diseases, OHSU, Portland, Oregon, United States of America; University of Kentucky, UNITED STATES

## Introduction

The viral replication cycle presents viruses with a series of logistical challenges: To be successful, the virus must synthesize new copies of its genetic material, induce the cell to translate its genome into protein, and coordinate the host and viral factors required to assemble new virus particles. Much of this occurs at intracellular membranes, and lipid bilayers are expected to both play a central role in organizing the molecular machinery required for each stage of virion biogenesis and provide a structural foundation for particle assembly. These functions are prominently exploited by viruses with single-strand, positive-sense RNA genomes, which dramatically remodel intracellular membranes to form specialized replication complexes (RC) in the cytoplasm of infected cells [1]. While the architectural features of RC membranes have been extensively studied, the emerging discipline of lipidomics has yielded new insight into the ways in which positive-strand RNA viruses modulate host lipid metabolism to create lipid environments favorable to RC formation and viral replication.

## Flaviviridae: A growing family of human pathogens

Among the major contributors to the positive-strand RNA virus global disease burden are the flaviviruses, a genus of enveloped viruses that includes Yellow fever virus (YFV), Zika virus (ZIKV), Dengue virus (DENV), and West Nile virus (WNV) and is closely related to Hepatitis C virus (HCV) [2]. Despite high degrees of morphological and genomic similarity, flaviviruses display a remarkable variety of tropisms and possible clinical outcomes, from neurological dysfunction caused by WNV and ZIKV to vascular leakage and hemorrhage in severe cases of DENV and YFV infection [3]. The emergence and expansion of ZIKV and other flaviviruses into new populations, and association with novel pathologies like microcephaly, have lent urgency to efforts to understand the host–virus interactions leading to disease, many of which remain enigmatic. With no therapeutic treatments available and no vaccines for most flaviviruses, understanding the mechanisms of these interactions is an important avenue toward the development of new antiviral therapies.

## Flavivirus biogenesis occurs on modified ER membranes

**Fig 1:**
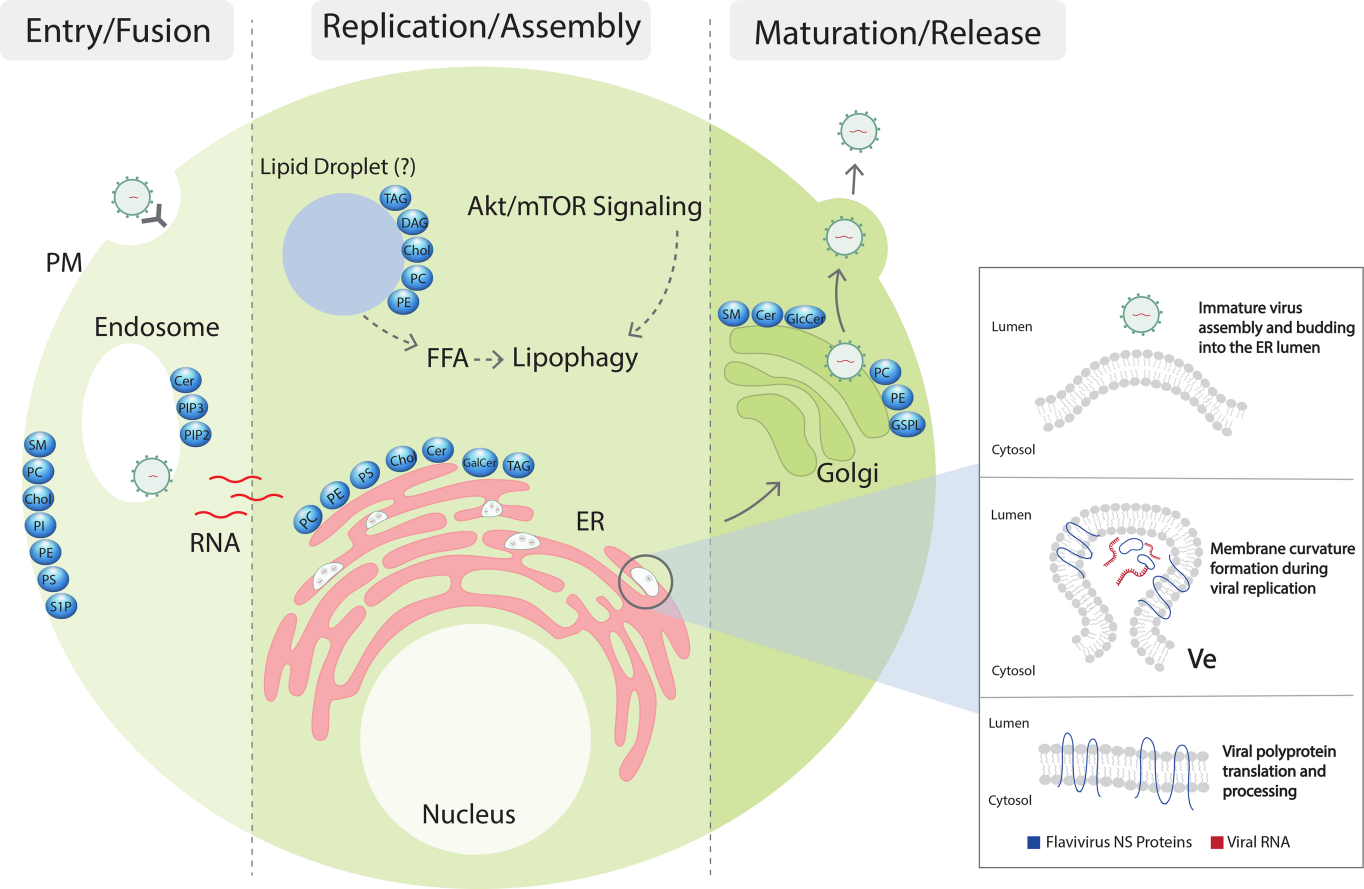
Flaviviruses modulate host lipids during infection. After flavivirus particles are internalized through receptor-mediated endocytosis, fusion with the membrane of the late endosome releases a single positive-sense RNA genome into the cytoplasm of the host cell. Translation by cellular ribosomes results in membrane-associated structural and nonstructural proteins, which curve ER lipid bilayers into invaginated replication vesicles and budding sites (inset). To facilitate membrane remodeling and replication, flaviviruses manipulate multiple aspects of both structural and bioactive lipid classes in an organelle-dependent manner. The resulting dysregulation of cellular pathways may contribute to cell death and clinical disease. Fully assembled virus particles are transported to the Golgi apparatus, where they undergo a maturation process and are released through exocytosis. Cer, ceramide; Chol, cholesterol; DAG, diacylglycerol; ER, endoplasmic reticulum; FFA, free fatty acids; GalCer, galactosylceramide; GlcCer, glucosylceramide; GSPL, complex glycosphingolipids; mTOR, mammalian target of rapamycin; NS, nonstructural; PC, phosphatidylcholine; PE, phosphatidylethanolamine; PIP2, phosphatidylinositol-(4,5)-bisphosphate; PI, phosphatidylinositol; PIP3, phosphatidylinositol-(3,4,5)-trisphosphate; PM, plasma membrane; PS, phosphatidylserine; S1P, sphingosine-1-phosphate; SM, sphingomyelin; TAG, triacylglycerol; Ve, invaginated vesicles.

Flavivirus particles are internalized through receptor-mediated endocytosis, followed by pH-mediated fusion, uncoating, and release of the RNA genome from the endosome into the host cytoplasm. This strand of positive-sense RNA is translated by host ribosomes to a single polyprotein, which is cleaved by cellular and viral proteases into three structural (capsid [C], membrane [prM], and envelope [E]) and seven nonstructural (NS) proteins that form the genomic replication machinery. A combination of membrane-associated NS proteins and co-opted host factors then collaborate to bend the surrounding endoplasmic reticulum (ER) membrane into clusters of invaginated vesicles (Ve) connected to the cytosol by a single pore [4–6] (Fig 1). A number of structural and immunofluorescence microscopy studies have shown colocalization of NS proteins and the double-stranded RNA (dsRNA) intermediate of viral replication with Ve membrane invaginations, indicating that replication occurs within the Ve lumen [7]. Additionally, envelopment of the flavivirus replication complex by host lipids shields dsRNA from cytoplasmic sensors of the innate immune system, contributing to pathogenesis [8]. Though the details of genome encapsidation and budding are less understood, the presence of large arrays of virus particles within the lumen of the ER, as well as putative budding sites located adjacent to Ve pore openings, have contributed to a model in which viral RNA is trafficked through the Ve pore to assembly sites on nearby ER membranes [9, 10]. Central to the budding process is membrane curvature induced by prM and E, which form a transmembrane heterodimer in immature virions [11, 12]. A scaffold of prM–E oligomers envelopes the flavivirus nucleocapsid and buds into the lumen of the ER, a procedure with clear parallels to cellular vesicle trafficking [13]. Strikingly, expression of prM–E causes membrane curvature and budding even in the absence of other viral proteins, leading to the production of subviral particles (SVPs) similar in structure to infectious virions [14]. Following budding, flavivirus particles undergo maturation in the Golgi apparatus and are released through exocytosis [15].

## Replication complex assembly remakes the host lipid landscape

Inducing the negative membrane curvature required for vesicle formation is a thermodynamically unfavorable process [16]. To lower this energy cost, a number of intracellular trafficking pathways modify the lipid composition of target membranes to increase the presence of lipids with properties, such as head group size or the number of unsaturations, that contribute to spontaneous curvature [17, 18]. Lipidomic analyses of cells infected with flaviviruses have shown that viral replication triggers significant changes in global lipid profiles, suggesting similar mechanisms may be required for viral membrane remodeling [19–22]. Studies performed both in model membranes and cultured cells have suggested that ceramide, a sphingolipid that is synthesized primarily in the ER, can play a vital role in the formation of membrane curvature and membrane vesiculation [23]. In line with these observations, WNV and DENV have been shown to increase levels of cellular ceramide and other sphingolipids during infection [20]. Whether other flaviviruses also alter sphingolipid metabolism remains to be established.

Similar results have been shown for phosphatidylcholine (PC), a second lipid class strongly enriched during flavivirus infection [19–22]. Remarkably, there appears to be substantial variation in up-regulation among PC species with different acyl-chain lengths and degrees of unsaturation, with longer, more saturated chains generally favored by WNV [20]. As with ceramide, enrichment of unsaturated PC has been implicated in vesicle formation by trafficking pathways [18]. Together, these lipidomics studies reveal a broad and flavivirus-specific modulation of host lipid metabolism, resulting in host membranes with lipid profiles favorable to RC remodeling and viral replication.

## Flaviviruses modulate fatty acid and cholesterol biosynthesis

In concert with changes in structure and lipid makeup, the ER undergoes significant expansion during flavivirus infection [5]. This growth coincides with the fractionation of lipid droplets (LD), ER-derived organelles that serve as cellular reservoirs of triglycerides and cholesterol [5, 24]. DENV activates an autophagy-dependent form of lipophagy that breaks down LD triglycerides to free fatty acids (FFAs), which are subjected to beta-oxidation in the mitochondria to generate ATP [24]. Simultaneously, DENV and WNV appear to increase de novo production of FFAs by redistributing the fatty acid synthase complex (FASN) to replication sites and up-regulating its activity [25–27]. Additional evidence for the reliance of DENV production on the content of LDs comes from a study showing recruitment of the DENV C protein to LD protein markers and pockets of neutral lipids, disruption of which impairs replication and assembly [28]. These interactions are similar to the association of LDs and HCV core protein, a direct cause of lipid accumulation and steatosis in chronically infected patients [29]. Thus, LD lipids may be required for multiple aspects of flavivirus infection and pathogenesis, from providing the raw materials and energy for replication to supporting efficient encapsidation and assembly of viral particles.

Fatty acid synthesis is regulated by sterol regulatory element-binding proteins (SREBPs), a family of membrane-bound transcription factors also responsible for controlling cholesterol biosynthesis [30]. Although total cellular cholesterol levels are only modestly perturbed in DENV-infected cells, inhibition of cholesterol metabolism results in significant decreases in DENV replication [31, 32]. A microscopy study has shown that WNV redistributes cellular pools of cholesterol from the plasma membrane to RCs and boosts the activity of 3-hydroxy-3-methylglutaryl-coenzyme A reductase (HMGCR), the rate-limiting enzyme in cholesterol biosynthesis [33]. Enrichment of cholesterol at sites of particle assembly may have an important effect on virion infectivity, as cholesterol in the DENV envelope is required for genome release after entry [34]. Along with sphingolipids, cholesterol is an important component of lipid rafts [35], raising the possibility of complementary cross talk between the diverse lipid pathways manipulated by flaviviruses.

## Flavivirus tropism, lipids, and disease

Mounting evidence suggests that different flaviviruses have different cellular and tissue tropisms with distinctive disease pathology. Although the exact determinants of differential flavivirus tropism are unknown, the lipid microenvironment of the tissue may have a critical role in this process. For instance, it is now well established that ZIKV crosses the human fetal–placental barrier to infect the developing central nervous system to cause microcephaly [36]. ZIKV infects human neural progenitor cells with far greater efficiency than other flaviviruses and causes uniquely deleterious effects during infection [37, 38]. The myelin sheath that surrounds central and peripheral neuronal cells is mainly composed of sphingolipids and cholesterol, and brain tissue is one of the richest in lipid content. Whether the lipid content of these neuronal cells plays a role in determining the tropism and pathology of ZIKV is yet to be determined.

An important process involved in the regulation of lipid metabolism that is affected by ZIKV and possibly by other flaviviruses is the Akt–mTOR signaling pathway [39]. ZIKV replication suppresses Akt–mTOR signaling, an outcome associated with microcephaly in infants with loss-of-function mutations [40]. Importantly, Akt–mTOR signaling interacts with the same autophagy pathways manipulated by DENV and WNV to induce lipophagy. How the cellular stress responses linked to microcephaly relate to ZIKV’s requirements for host lipids is an important question for future studies to address.

## Concluding remarks and future perspectives

Recent advances in the fields of genomics and proteomics have transformed our understanding of health and diseases. Despite the latest improvements in analytical approaches—in particular, liquid chromatography and mass spectrometry—the field of lipids lacks a corresponding advancement of knowledge in host–pathogen interactions. Due to the complexity of lipids and the absence of robust tools, manipulation of lipid composition in a controlled manner remains an experimental challenge. A combination of recently developed lipid tools such as the bi- and trifunctional lipid probes would provide the means to monitor the spatial and temporal distribution of cellular lipids during infection [41]. Combined with quantitative proteomics and lipid profiling, these tools would also provide a means to illuminate the “interactome” of a particular lipid during infection and reveal novel therapeutic targets for infectious diseases.
